# Pollux: platform independent error correction of single and mixed genomes

**DOI:** 10.1186/s12859-014-0435-6

**Published:** 2015-01-16

**Authors:** Eric Marinier, Daniel G Brown, Brendan J McConkey

**Affiliations:** David R. Cheriton School of Computer Science, University of Waterloo, 200 University Ave W, Waterloo, ON N2L 3G1 Canada; Department of Biology, University of Waterloo, 200 University Ave W, N2L3G1 Waterloo, Canada

**Keywords:** Error correction, Next-generation sequencing, Assembly

## Abstract

**Background:**

Second-generation sequencers generate millions of relatively short, but error-prone, reads. These errors make sequence assembly and other downstream projects more challenging. Correcting these errors improves the quality of assemblies and projects which benefit from error-free reads.

**Results:**

We have developed a general-purpose error corrector that corrects errors introduced by Illumina, Ion Torrent, and Roche 454 sequencing technologies and can be applied to single- or mixed-genome data. In addition to correcting substitution errors, we locate and correct insertion, deletion, and homopolymer errors while remaining sensitive to low coverage areas of sequencing projects. Using published data sets, we correct 94% of Illumina MiSeq errors, 88% of Ion Torrent PGM errors, 85% of Roche 454 GS Junior errors. Introduced errors are 20 to 70 times more rare than successfully corrected errors. Furthermore, we show that the quality of assemblies improves when reads are corrected by our software.

**Conclusions:**

Pollux is highly effective at correcting errors across platforms, and is consistently able to perform as well or better than currently available error correction software. Pollux provides general-purpose error correction and may be used in applications with or without assembly.

## Background

The introduction of second-generation sequencing technologies has enabled inexpensive, high-throughput sequencing. These technologies typically produce millions of short DNA sequences (reads). These reads contain a non-trivial number of errors which complicate sequence assembly [1,2] and other downstream projects which do not use a reference genome. The frequency and type of errors depends largely on the sequencing technology, but also on the composition of the target being sequenced. These errors complicate *de novo* assembly by hiding true contig and scaffold connections while enabling false connections. Furthermore, they mislead analyses by creating an inaccurate or incomplete picture of the data.

The error correction problem involves identifying and correcting errors in reads introduced during nucleotide sequencing. The number and type of errors in a set of reads depends primarily on the sequencing technology employed and the number of sequenced bases, but also on the true frequency of error prone regions such as homopolymers. The error types common to all methods are substitution, insertion, and deletion. A more specific sequencing error is a homopolymer region being miscalled in its length, resulting in spurious insertions or deletions of the repeated nucleotide. Substitution, or mismatch, errors are single base errors where one base is replaced by another. Insertion errors are erroneous bases inserted into the sequence, and are corrected by deleting the erroneous bases. Conversely, deletion errors are bases removed from a sequence, and are corrected by inserting the removed bases back into the sequence. The error types and rates of Illumina MiSeq, Ion Torrent PGM, and Roche 454 are varied as a consequence of the differences in sequencing technology.

Illumina reads contain substitutions as the dominant error type. MiSeq has reported total error rates ranging from substitution rates of 0.1% and indel rates of under 0.001% [3], to 0.80% total error rate [4]. MiSeq errors are not uniformly distributed across the genome [5], but appear to be more frequent around homopolymer runs [4,6], GGC triplets [5], or towards the 3’ ends of reads [7]. There appears to be a higher frequency of mismatches within 10 bases downstream of both a GGC triplet in the forward direction and its reverse complement (GCC) in the reverse direction. However, there seems to be no correlation between the GGC triplet and a higher mismatch rate if the following triplet is AT-rich [4]. Furthermore, these errors seem to represent as little as 0.0015% of bases [8]. Interestingly, Luo *et al.* report homopolymer associated indels with Illumina Genome Analyzer II in 1% of genes reported from assembly, suggesting homopolymers may introduce more errors in Illumina data than previously expected.

Errors in Roche 454 and Ion Torrent technologies are typically miscalled homopolymer run lengths. Ion Torrent PGM appears to have a higher error rate for calling homopolymers of any length than Roche 454 GS Junior [4]. Ion Torrent PGM has reported total error rates at 1.71% [4] and indel rates of 1.5% [3]. The accuracy of PGM reads appears to steadily decrease towards the end of the read [3]. Roche 454 GS Junior has reported total error rates of 0.5% [8] and indel rates of 0.38% [3]. Luo *et al.* report a homopolymer error rate bias with Roche 454 FLX Titanium reads within AT-rich homopolymers. Total homopolymer error rates were as high as 25% for homopolymers of length 7 and nearly 70% for homopolymers of length 11.

When considering the overall coverage of the sequencing project, Illumina MiSeq appears to be largely unaffected by GC content while Ion Torrent PGM appears to have more uneven coverage [4]. PGM reads favour GC-rich content, and the effect is particularly severe when sequencing AT-rich genomes. PGM sequencing of the AT-rich *P. falciparum* resulted in no coverage for approximately 30% of the genome. The consequence of this is that Ion Torrent PGM read depths may have a greater deviation from a random sampling process than would be expected for Illumina MiSeq. Loman *et al.* assemble MiSeq, PGM, and GS Junior *E. coli* reads and align contigs to a reference of the same *E. coli* isolate. They showed that GS Junior reads align in the greatest proportion with 3.72% of reads unaligned, followed by MiSeq at 3.95% and PGM at 4.6%.

We introduce an error correction tool capable of correcting errors introduced by Illumina, Ion Torrent, and Roche 454 sequencing technologies. We approach the problem of error correction conservatively, removing little information and requiring a high degree of confidence to make a correction. Our program Pollux first scans across all reads, divides reads into *k*-mers of length 31 (default), and counts the number of occurrences of each observed *k*-mer. It then scans reads a second time, generates a *k*-mer depth profile for each read, and uses this information to correct the *k*-mer profile. Specific *k*-mers are not flagged as erroneous as a result of occurring infrequently, but rather are explored when a discontinuity in *k*-mer frequencies is observed when transitioning from one *k*-mer to the next in a given read. The software is sensitive to low-coverage reads and does not favour high-coverage reads. In contrast to other methods [9], we do not specifically use quality scores for error correction. However, the software updates the quality of corrected bases to reflect the improved confidence of a base after correction. The software can easily be integrated into a larger pipeline analysis. It improves the utility of reads and can improve hybrid assembly, as it can correct reads generated from a variety of sequencing technologies. It may also improve sequence analysis projects that may not require assembly, such as metagenome analysis [10].

## Implementation

**Figure 1:**
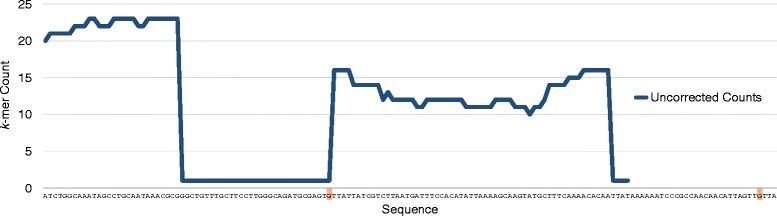
**The** ***k*** **-mer counts associated with an Illumina MiSeq read containing two highlighted substitution errors.** The *k*-mers are of length 31. A data point corresponds to the number of times a left-anchored *k*-mer, starting at a given position within the sequence, has been observed in the entire read set. The first error is located near the middle of the read and affects the counts associated with 31 *k*-mers. The second error located only four positions in from the 3’ end of the read and affects only 4 *k*-mer counts.

Similar to other methods [9,11], we approach the problem of error correction using *k*-mers, consecutive *k*-letter sequences identified in reads. However, our approach does not identify individual *k*-mers as erroneous, but rather compares the counts of adjacent *k*-mers within reads and identifies discontinuities. These discontinuities within reads are used to find error locations and evaluate correctness. We decompose a read into its *k*-mers and calculate their associated *k*-mer counts (Figure 1), which are the number of times a given *k*-mer has appeared in the entire set of reads. We use default *k*-mer lengths of 31, which are slightly larger than typically used in assembly [12,13]. We choose to use longer *k*-mers because this lets us avoid common short repeats which might otherwise confound our correction procedure. We choose *k* = 31, because it is the longest odd *k* that can be represented in a 64-bit word. We only record *k*-mers observed in the data set, so we maintain an extremely small subset of all possible *k*-mers of length 31. A read that is not erroneous is assumed to have a *k*-mer count profile that is reflective of a random sampling process, given local coverage. In contrast, a read that contains an error is likely to have *k*-mer counts that deviate unexpectedly from this random process. A substitution error, located at least *k* bases away from the ends of the read, will result in *kk*-mer counts affected. If this error is unique within the read set, these counts will drop to 1. A similarly defined insertion error will affect *k*+*nk*-mer counts and a deletion error will affect *k*−*n* counts, where n is the length of the indel. These unexpected drops in *k*-mer counts to a low depth are often erroneous, but we do not immediately make this assumption.

We recognize the boundaries of adjacent *k*-mers which deviate unexpectedly and identify nucleotide positions associated with the discrepancy. We then explore the space of possible corrections and evaluate the fitness of these corrections. We choose the correction that removes or minimizes the *k*-mer count discontinuity. If there exist multiple corrections which achieve this, we choose the correction that improves the most *k*-mer counts maximally beyond the current erroneous location. This is particularly important when correcting reads that contain multiple errors or within reads that may be corrected in multiple ways. We attempt to correct the read such that the *k*-mer counts associated with it are more reflective of changes in *k*-mer counts generated from a random sampling process. This approach allows for a sensitive error correction strategy regardless of local depth of sequencing for the read of interest. However, homopolymer errors are frequently not unique and require a special acceptance criteria as their correct *k*-mer counts after correction often still appear erroneous. Pollux corrects each read independently and does not update recorded *k*-mer counts as a result of correction. The outcome of correction does not depend on the order of the reads.

This approach to correction may be complicated by the boundaries of repeated regions, which may also create *k*-mer count discontinuities. These boundaries will initially be flagged as potential errors. However, exploration of the error site will often reveal that no such error exists and the boundary region will be ignored. There is the possibility of false positive corrections within very low coverage regions containing a single nucleotide polymorphism when there exists a high coverage alternative in the data set. These low coverage regions will sometimes be corrected to their high coverage alternative, but this is relatively rare.

For implementation of the error correction procedure described above, we have made an effort to reduce execution times and memory requirements. For *k*-mer counting, we compress nucleotide information and operate within a two-bit alphabet. In combination with our hash-table strategy, this allows us to use relatively large *k*-mers. Furthermore, we process reads in batches of adjustable size. As a result, the memory limitation is a consequence of the hash table when counting all *k*-mers. We attempt to reduce the size of the hash table by removing all unique *k*-mers before correction.

Another consideration is choosing a *k*-mer count evaluation window that balances true and false positives during error correction. An evaluation criteria that requires a single correction to improve all *k*-mer counts spanning the error location will avoid many false positives, but will often miss true positive corrections in reads containing multiple errors. On the other hand, an evaluation criteria which requires a correction to improve only one *k*-mer count will introduce many false positives by correcting errors inappropriately, such as correcting a substitution error with an insertion, which instead propagates the error forward. We use an evaluation window which considers the fewest amount of *k*-mers required to make a confident correction while avoiding error propagation. This requires evaluating multiple *k*-mers containing bases following the erroneous base to ensure our correction is appropriate. This allows Pollux to correct multiple, although non-adjacent, errors in close proximity.

### Error correction methodology

**Figure 2:**
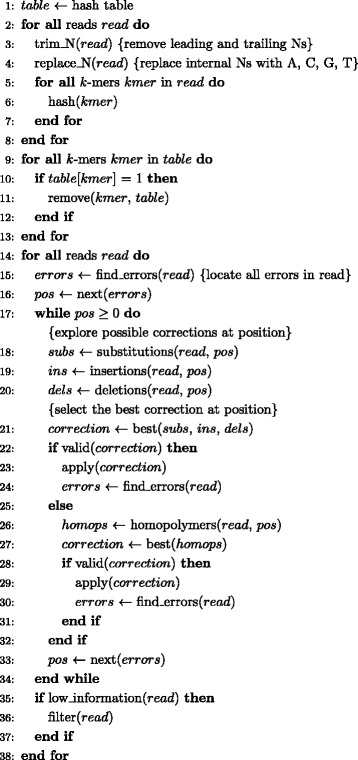
**Algorithm pseudocode.** A pseudocode for the error correction algorithm.

A pseudocode of our error correction procedure is outlined in Figure 2. We begin with a basic preprocessing step which removes all leading and trailing Ns. Internal Ns are replaced with either A, C, G, or T in a reproducible and evenly distributed manner. This allows us to operate within a four-character alphabet and treat internal Ns as substitution errors. We then construct a hash table of all *k*-mers in the set of reads. This involves maintaining a count of all observed *k*-mers and their reverse complements as separate entries in a single hash-table.

After aggregating *k*-mer information from all reads, we free significant amounts of memory by removing all unique *k*-mers from our hash table and implementing a policy of reporting a count of 1 whenever a *k*-mer is not found in our hash table. Removing these *k*-mers significantly reduces memory requirements and improves execution times for the error correction procedure. In one *E. coli* data set [3], 41% of Illumina MiSeq, 85% of Ion Torrent PGM (1), and 46% of Roche 454 GS Junior (1) *k*-mers are unique. These *k*-mers contribute no additional information and can be safely removed using this strategy. The number of unique *k*-mers will depend on sequencing depth, technology, and the number of error prone regions.

We construct a *k*-mer count array associated with each read and fill each entry with the count of the *k*-mer that is left-anchored at that index position in the sequence. *r*−*k*+1 such counts associated with a given read, where *r* is the length of the read and *k* is the length of the *k*-mers. Reads of length shorter than *k* are left uncorrected. We scan across the array and observe changes in *k*-mer counts. When we observe an unexpected change in counts between consecutive *k*-mer counts in a read, we locate the nucleotide position associated with the discrepancy and identify the position as a potential error source. We do not identify reads containing low *k*-mer counts to be erroneous if such counts appear to follow a random sampling process with no discontinuities in *k*-mer depth. A more detailed description of this procedure follows. We define consecutive *k*-mer counts to be potentially erroneous if their difference is larger than a specified threshold. This threshold requires consecutive *k*-mer counts to have a difference of greater than 3 and greater than 20% the larger count to be flagged as a possible error. This threshold is quite sensitive, and will identify discontinuities corresponding to homopolymer repeats as well as substitution and indel errors. It additionally works quite well for high coverage and medium coverage sequencing projects. The fixed-number component of the threshold operates when correcting low to medium coverage regions while the percent-based component operates during medium to high coverage correction. The thresholds are designed to be conservative in high coverage and operate well in moderate coverage. At low coverage the rate of recognizing errors is reduced, as less information is available for error calling. We found that when correcting the same *E.coli* MiSeq data set [3], we were able to correct 94% of errors at 50x mean coverage but this was reduced to 65% of errors at 5x mean coverage.

We determine the erroneous nucleotide position *N* to be *N*=*d* if we observe a low-to-high *k*-mer count discrepancy and *N*=*d*+*k* if we observe a high-to-low discrepancy, where *d* is the left index of the discrepancy. This is applicable for substitution, insertion, deletion, adjacent, and homopolymer errors. In the case of deletion errors, a low-to-high *k*-mer count discrepancy will point to the base immediately before the deletion and a high-to-low discrepancy will point to the base immediately following. With respect to homopolymers, the leftmost base in a homopolymer run is used as an anchor during correction and is found by scanning left from *N*+1 in the case of a low-to-high discrepancy and *N*−1 with a high-to-low discrepancy. The exact position of a non-homopolymer adjacent error will depend on the type of adjacent errors. However, this procedure will locate the first problematic base adjacent to the high *k*-mer count region being considered.

We choose to evaluate *k*-mer counts such that the evaluation *k*-mers overlap largely with the region of the read which appears to contain no errors. This approach allows us to perform multiple corrections within close proximity. The very first *k*-mer we choose to evaluate is the *k*-mer that entirely overlaps a trusted region of the read and borders the erroneous region. We define a region to be trusted if it contains no *k*-mer count discrepancies within it. Additional evaluation *k*-mers extend into the erroneous region. In order for a correction to be accepted, it must improve the counts of multiple evaluation *k*-mers up to at least the *k*-mer which contains the first base immediately following the erroneous base. We require information about the region after the erroneous base to prevent propagating an error further down the read by performing misleading substitutions or insertions. However, we do not correct adjacent errors outside of homopolymer errors, which are evaluated separately. We simultaneously consider all possible substitution, insertion, and deletion corrections within a single read. The correction selected is the one that produces *k*-mer counts that improve the most evaluation *k*-mers. We note that our library of *k*-mer counts is not updated as a consequence of a correction so the order of correction has no effect. The correction procedure is repeated multiple times within a read until either there remains no detected errors which are correctable or we determine we have made too many corrections. In the later case, we revert all changes and do not correct the read.

We observe the *k*-mer counts in a read after completing corrections and flag any reads containing more than 50% unique *k*-mers (i.e. *k*-mer depth of 1), as such reads are typically of poor quality. In the case of paired-end read corrections, we only flag reads if both pairs meet this criteria. This approach enables us to correct many unusable reads which might otherwise be discarded and remove reads which appear to contribute little information. We do not consider quality scores when making corrections or removing reads. However, we modify quality scores to be the average of the quality scores adjacent to corrected base to enable downstream processing. No assumptions are made about the accuracy of quality scores for different sequencing technologies. Furthermore, one benefit of avoiding quality scores is our software is not affected by chimeric sequences or by reads which contain misleading quality scores.

### Homopolymer corrections

Homopolymer errors differ from other error types in that they frequently coincide and often contain multiple adjacent errors. This causes the *k*-mer counts associated with a read containing an accurate homopolymer length to often appear discontinuous and *k*-mer count discontinuities associated with erroneous homopolymer lengths to often appear less severe than their counterparts. This is particularly common for very long homopolymers. We observe however that homopolymer errors tend to have a recognizable distribution of *k*-mer counts associated with varied homopolymer lengths. The accurate-length homopolymers tend to have the largest *k*-mer counts associated with them and as the deviation in length from the correct value increases, the associated *k*-mer counts tend to decrease. When we detect a possible homopolymer error, we explore a range of homopolymer lengths and select the length which maximizes a specific subset of *k*-mer counts. We evaluate the two *k*-mers that overlap primarily the trusted region, the entire homopolymer, and the two bases immediately following the homopolymer run. Since we have observed homopolymers being erroneously reported as length 1, we include the possibility of single nucleotide homopolymer errors (e.g. where a homopolymer of length 2 is reported as length 1). In all case, we consider possible lengths from one half to twice the initial length. Pollux performs homopolymer corrections independently and only after all other correction possibilities have been exhausted. As the homopolyer correction algorithm is more forgiving, it would otherwise possible to mistake random noise as a slight improvement and miss the true correction.

The majority of homopolymer corrections we make are adjustments that modify the length of the homopolymer by 1. When we correct an *E. coli* data set [3], we see that 85% of GS Junior (1) and 92% of PGM (1) homopolymer corrections are adjustments of length 1. Ion Torrent PGM had a higher rate of homopolymer-associated indels per base than Roche 454 GS Junior. We reported making 0.018 indel corrections per base in PGM (1) and 0.0034 indels per base in GS Junior (1). Loman *et al.* report 0.015 indels per base in PGM and 0.0038 indels per base in GS Junior, which is consistent with our results.

## Results

We use data from the Loman *et al.* [3] benchtop sequencing comparison study to evaluate how well our software performs by mapping corrected and uncorrected reads to the corresponding reference genome. The data consists of read sets from Roche 454 GS Junior (SRA048574), Ion Torrent Personal Genome Machine (SRA048511), and Illumina MiSeq (SRA048664) technologies generated from the same *E. coli* O104:H4 isolate, which was the source of a food poisoning outbreak in Germany in 2011. The authors provide a reference genome constructed from reads generated by a Roche 454 GS FLX+ system. These reads had a modal length of 812 bases and over 99% of sequenced bases were Q64 bases. Additionally, a paired end library with 8kb inserts was generated to assist with assembly.

The reference *E. coli* genome consists of multiple scaffolds corresponding to the bacterial chromosome and two large plasmids. The authors note that the large plasmids and the Shiga toxin-producing phage have significantly higher sequence coverage than the bacterial chromosome within the Illumina and Ion Torrent data sets. In particular, there is 25-fold coverage of the chromosome and 625-fold coverage of the plasmids in the Illumina data set. This amounts to approximately half the reads mapping to the large plasmids. This effect is much less pronounced in the Ion Torrent data sets.

We use SMALT (www.sanger.ac.uk/resources/software/smalt/) to align uncorrected and corrected reads to the reference scaffolds. A summary of our software’s reported corrections can be found in Table 1. As homopolymer repeat errors of two or more nucleotides are extremely infrequent in Illumina data, we disable homopolymer corrections when correcting Illumina data. This avoids introducing a small number of homopolymer associated errors. A total of 99% of the GS Junior (1), 89% of the PGM (1), and 99% of the MiSeq reads are retained as high quality after correction. The filtered low information reads are corrected, but are separated from the high information reads. A custom Python script is used to aggregate information from the SMALT alignment about corrected, missed, and introduced errors. We observe whether a read is aligned or unaligned to the reference and whether the reference scaffold it is aligned with has changed. During the assessment procedure, we discard incomparable results such as read pairs which do not align to the same reference scaffold or have alignment starting positions further than twenty bases apart (e.g. reads aligning to similar repetitive regions). Similar to Loman *et al.*, we ignore soft-clipped alignment regions and we additionally ignore all aligned bases which are not contained within the mutual alignment interval of the pair of reads with respect to the reference. These removal processes leave us with 99% of the GS Junior (1), 95% of the PGM (1), and 94% of the MiSeq aligned bases for analysis. We create a list of errors in both reads determined by their error type (mismatch, insertion, or deletion) and by their position with respect to the reference. We consider a single nucleotide alignment with an N to be erroneous when the N is located only within the read and not erroneous when located within the reference. When an error is found in an uncorrected read, but not in its corresponding corrected read, we report a corrected error. Conversely, when an error is found in a corrected read, but there is no such error in the corresponding uncorrected read, we report an introduced error. If the same error appears in both the uncorrected and corrected reads, we report it as an uncorrected error. The results of the alignment comparisons are found in Table 2.

**Table 1:** **The number of corrections reported and low-information reads removed by Pollux**

	**Number**	**Total number**	**Corrections**				**Reads**
**Platform (Run)**	**of reads**	**of bases**	**Mismatches**	**Insertions**	**Deletions**	**Homopolymers**	**removed**
454 GS Junior (1)	135,992	70,999,968	24,100	164,198	56,144	17,221	1%
454 GS Junior (2)	137,528	71,710,564	21,004	167,999	53,947	13,535	1%
Ion Torrent PGM (1)	2,483,868	303,579,279	610,872	2,609,205	1,863,522	1,106,908	11%
Ion Torrent PGM (2)	2,154,577	260,017,346	561,024	2,215,086	1,765,495	968,961	13%
MiSeq	1,766,516	250,356,566	250,896	1,893	3,349	0	1%

All reads are sequenced from the same O104:H4 *E. coli* isolate. Substitution, insertion, deletion, and homopolymer corrections are performed on all data sets except for MiSeq, for which we do not perform homopolymer corrections. The percentage of reads which were removed as a consequence of more than 50% unique *k*-mers is provided under *Reads Removed*.

**Table 2:** **Alignment comparison of corresponding uncorrected and corrected reads against the reference genome**

	**Corrected (Abundance (counts/kb))**					**Introduced (Abundance (counts/kb))**			
**Platform (Run)**	**Total**	**Mismatches**	**Insertions**	**Deletions**		**Total**	**Mismatches**	**Insertions**	**Deletions**
GS Junior (1)	**81%**	76% (0.33)	82% (2.28)	81% (0.81)		**4%**	11% (0.048)	2% (0.065)	6% (0.063)
GS Junior (2)	**85%**	79% (0.24)	86% (2.23)	83% (0.76)		**4%**	15% (0.044)	2% (0.059)	6% (0.050)
PGM (1)	**88%**	82% (1.68)	91% (7.67)	86% (7.10)		**3%**	2% (0.046)	2% (0.16)	5% (0.44)
PGM (2)	**86%**	80% (1.72)	90% (7.47)	84% (7.70)		**4%**	2% (0.052)	2% (0.16)	6% (0.52)
MiSeq	**94%**	95% (0.85)	10% (0.0015)	78% (0.007)		**1%**	1% (0.010)	4% (0.0007)	8% (0.0007)

All reads are sequenced from the same O104:H4 *E. coli* isolate. Corresponding uncorrected and corrected reads are aligned to the reference genome using SMALT. Incomparable alignments are removed. Corrected errors reflect alignment errors which are found in uncorrected reads but not in corrected reads. Similarly, introduced errors are a consequence of alignment errors found in corrected reads but not in uncorrected reads.

We correct the majority of errors within all data sets and corrections are sequencing technology appropriate. Additionally, we introduce very few errors with respect to the number of errors in the uncorrected reads. We correct 86% of insertion and 83% of deletion errors in the GS Junior (2) data set while only introducing 2% more insertions and 6% more deletion errors; that is, we correct about 20 errors for every 1 error introduced. We correct 91% of insertion and 86% of deletion errors in the PGM (1) data set while introducing 2% more insertion and 5% more deletion errors. We report 95% of substitution errors corrected in our MiSeq (1) data set while introducing only 1% more of such errors. Overall, we correct 85% GS Junior (2), 88% PGM (1), 94% MiSeq errors, and introduce under 4% new errors in all data sets. We appear to have some difficulty correcting MiSeq insertion errors with only 10% of MiSeq insertion errors corrected. However, these insertion errors make up only 0.2% of the total, and may also include insertion errors present within the 454 GS FLX+ reference assembly. Additionally, we introduce 15% more substitution errors in GS Junior (2), but the overall number of errors introduced in the data set is 4%.

There are a number of issues which should be considered when interpreting the results. The reference genome is sequenced using Roche 454 GS FLX+ and it will contain some errors. This will give the MiSeq data the appearance of having higher than expected amounts of uncorrected indel errors. Additionally, a small number of corresponding uncorrected and corrected reads may produce equal-scoring alignments which differ only slightly. When these alignments contain an error which is resolved differently in each alignment, the error may appear to be corrected and reintroduced. However, we expect alignment noise to be minimal since we both the reads and the reference are sequenced from the same *E. coli* isolate and the reference is of high quality. Furthermore, since our software attempts to find corrections which improve *k*-mer counts maximally, it is possible to report compound errors as an alternative error type. For example, a homopolymer with a single insertion adjacent to a homopolymer with a single deletion may appear as a mismatch and be corrected as such. This does not have an adverse affect on the correction itself, but may result in reporting more substitution corrections than expected.

### Comparison

We compare our software to several other error correctors. These include Quake [9], SGA [14], BLESS [15], Musket [16], and RACER [17]. We use the Roche 454 GS Junior (1), Ion Torrent PGM (1), and Illumina Miseq *E. coli* sequencing data sets available in the Loman *et al.* comparison [3]. Additionally, we include a *S. aureus* Illumina Genome Analyzer II data set (SRX007714, SRX016063) available in GAGE [2] as well as *L. pneumophila* (SRR801797) and *M. tuberculosis* (ERR400373) Illumina HiSeq data used to previously benchmark error correction [18]. We evaluate the effect of correction in the same manner as described above. The results of this comparison are shown in Table 3. We use *k* = 31 for all software except Quake, which uses *k* = 19 because of hardware memory limitations. However, we note Quake specifies using *k*-mers of approximately this size [9]. We additionally note that Quake, SGA, and Musket were intended to only correct Illumina sequencing data. However, we include Roche 454 and Ion Torrent corrections for completeness. Pollux, GAGE, and SGA perform read filtering whereas BLESS, Musket, and Racer do not filter reads.

**Table 3:** **Comparison of various error correction software**

	**Illumina MiSeq - E. coli**			
	**Errors**	**Errors**	**Reads**	**Run**
**Software**	**corrected (%)**	**introduced (%)**	**removed (%)**	**time (min)**
Pollux	**93.81**	1.28	0.89	3.01
Quake	58.78	0.05	0.92	4.09
SGA	78.65	0.09	1.11	15.51
BLESS	83.21	0.10	0.00	0.86
Musket	81.75	0.15	0.00	2.37
RACER	86.59	1.60	0.00	1.07
	**Illumina genome analyzer II - S. aureus**			
	**Errors**	**Errors**	**Reads**	**Run**
**Software**	**corrected (%)**	**introduced (%)**	**removed (%)**	**time (min)**
Pollux	**87.04**	0.38	31.73	3.67
Quake	75.30	0.10	29.81	4.81
SGA	47.45	0.02	10.71	14.28
BLESS	55.32	0.06	0.00	0.49
Musket	45.04	0.14	0.00	6.96
RACER	75.76	0.28	0.00	0.68
	**Illumina HiSeq - L. pneumophila**			
	**Errors**	**Errors**	**Reads**	**Run**
**Software**	**corrected (%)**	**introduced (%)**	**removed (%)**	**time (min)**
Pollux	96.16	0.13	6.01	14.40
Quake	**99.66**	0.00	4.25	22.25
SGA	84.61	0.03	3.87	73.82
BLESS	87.61	0.03	0.00	3.56
Musket	83.33	0.10	0.00	18.67
RACER	94.09	0.16	0.00	4.14
	**Illumina HiSeq - M. tuberculosis**			
	**Errors**	**Errors**	**Reads**	**Run**
**Software**	**corrected (%)**	**introduced (%)**	**removed (%)**	**time (min)**
Pollux	**69.98**	0.83	6.51	4.24
Quake	68.06	0.12	3.06	5.62
SGA	31.99	0.16	0.46	17.75
BLESS	60.01	0.13	0.00	1.04
Musket	44.68	0.80	0.00	5.93
RACER	65.14	1.28	0.00	0.79
	**Roche 454 GS Junior - E. coli**			
	**Errors**	**Errors**	**Reads**	**Run**
**Software**	**corrected (%)**	**introduced (%)**	**removed (%)**	**time (min)**
Pollux	81.02	4.14	0.82	5.45
Quake	0.21	0.00	0.07	3.62
SGA	8.94	3.63	1.64	5.33
BLESS	34.68	1.27	0.00	0.24
Musket	0.00	0.00	0.00	0.05
RACER	**82.03**	24.27	0.00	0.36
	**Ion torrent PGM - E. coli**			
	**Errors**	**Errors**	**Reads**	**Run**
**Software**	**corrected (%)**	**introduced (%)**	**removed (%)**	**time (min)**
Pollux	**87.83**	3.43	10.90	26.75
Quake	12.35	2.03	37.60	11.11
SGA	5.43	1.12	0.16	55.93
BLESS	22.82	0.52	0.00	1.25
Musket	9.40	4.88	0.00	47.27
RACER	67.86	15.95	0.00	1.64

The evaluation is performed by aligning corresponding uncorrected reads and corrected reads, which were not removed, against a reference genome using SMALT. Corrected errors are an aggregate of all alignment errors which are found in uncorrected reads but not in corrected reads. Similarly, introduced errors are an aggregate of all alignment errors found in corrected reads but not in uncorrected reads and are relative to the sum of corrected and uncorrected errors. The percentage of reads removed by each software is noted. We note that Quake, SGA, and Musket were intended to only correct Illumina sequencing data.

We find that Pollux corrects the greatest percentage of errors in four of the six test sets and is second in the remaining two. Pollux filters reads with similar aggressiveness as Quake and SGA. RACER corrects the most errors within the GS Junior data, corrects the majority of errors in the PGM data, and performs well on Illumina data sets while filtering no reads. The amount of errors introduced by RACER is comparable to Pollux within the Illumina data. However, RACER introduces a significant number of errors within GS Junior and PGM data. SGA, BLESS, and Musket correct and introduce similar amounts of errors within Illumina data. These error correctors remove a significant number of errors while introducing extremely few errors. However, of these three, only BLESS performs well on GS Junior and PGM data. Quake performs exceptionally well on HiSeq *L. pneumophila*, correcting 99.66% of errors while introducing almost no additional errors. The effect of read filtering is significant within *S. aureus* data. Pollux is able to obtain a high percentage of errors corrected in this data because of its ability to remove reads which do not contribute information. This is supported by the observation that an assembly generated from corrected *S. aureus* reads improves significantly following correction.

### Mixed genome correction

Pollux is also intended for mixed genome data sets, such as those that would be obtained in a metagenomics study. We create a suitable high quality mixed reference data set for testing by incorporating two data sets from GAGE [2] with the Loman *et al.E. coli* reads [3]. This data set is comprised of uncorrected Illumina data from *E. coli* (SRA048664) [3], *S. aureus* (SRX007714, SRX016063) [2], and *R. sphaeroides* (SRX033397, SRX016063) [2] and is used to evaluate our software’s ability to correct errors in a mixed genome environment. The *E. coli* reads are the same as above and were sequenced with Illumina MiSeq. The *S. aureus* and *R. sphaeroides* reads were sequenced using an Illumina Genome Analyzer II. The *E. coli* reference was assembled from Roche GS FLX+ reads, while the *S. aureus* and *R. sphaeroides* references were assembled with reads generated from Sanger sequencing. As our error correction and evaluation procedures ignore the order of reads, we concatenated all read sets into a single file and similarly concatenated all references into a single reference file. The mixed data set was comprised of 35% *E. coli* reads, 25% *S. aureus*, and 40% *R. sphaeroides*. We correct these mixed reads using *k* = 31 with homopolymer correction disabled and evaluate the effect using the same alignment procedure described above.

An overall total of 82% of errors were reported corrected with only 0.6% more errors introduced. Specifically, our software corrected 82% of substitution errors, 70% of insertion errors, and 73% of deletion errors, with 98% of all of corrections being substitutions. A total of 19% of reads were removed using our *k*-mer removal criteria and not aligned. We evaluated 94% of aligned bases after discarding incomparable alignment locations and soft-clipped bases. While 19% seems substantial, we note that Quake [9] and the error correction procedure within ALLPATHS-LG [13] remove 37% and 36% of the *S. aureus* reads, respectively, and similarly removed 26% and 31% of the *R. sphaeroides* reads [2] when correcting the data sets independently.

### Assembly improvements

We evaluate our error correction software as a preprocessing step before *de novo* assembly. The *E. coli* Illumina MiSeq [3] and *S. aureus* Illumina Genome Analyzer II [2] read sets are used to evaluate the effect of our software on assembly when correcting paired reads with both short and long insert lengths. The *E. coli* reads are paired and have an average read length of 142. The *S. aureus* data set consists of paired fragment reads of length 101 with average insert lengths of 180 and long-range paired-end reads of length 37 with average insert lengths of 3500. The paired reads are corrected together using our software’s paired-end correction. However, we choose not to remove any short-jump *S. aureus* reads as nearly half of short-jump reads were flagged as having more than 50% unique *k*-mers. Removing these reads would have rendered much of the valuable short-jump information unusable.

Velvet [12] is used to assemble the *E. coli* and *S. aureus* Illumina data sets. We use assembler default settings (*k* = 31) for the *E. coli* reads and assembly settings as described by GAGE [2] (*k* = 31) for the *S. aureus* reads. The results of the assemblies can be found in Table 4. We compare the common assembly metrics number of scaffolds and N50 of assemblies using uncorrected and corrected reads. We additionally include NGA50, as calculated by QUAST [19], which represents the contig length such that equal or greater length contigs account for at least 50% the length of the genome. This value is calculated after breaking misassembled contigs and additionally differs from the N50 in that it is with respect to the genome size and not the assembly length. There are fewer scaffolds and larger N50 values in the error corrected assemblies than there are in there uncorrected counterparts. The uncorrected *E. coli* assembly produces 2120 scaffolds with an N50 of 31 kb and a maximum scaffold of size 99 kb. This improves to 1840 scaffolds with an N50 of 37 kb and a maximum contig of size 163 kb. The NGA50 improves slightly as well, increasing from 84 to 85 kb. The *S. aureus* assembly improves even more significantly. The uncorrected assembly produces 737 scaffolds with an N50 of 192 kb and a maximum scaffold size of 435 kb. The corrected assembly reduces the number of scaffold to 603 and has a N50 of 1771 kb, which is the maximum scaffold size, and is longer than half the genome length. Furthermore, the NGA50 improves substantially after correction, increasing from 145 kb to 202 kb.

**Table 4:** **Comparison of** ***de novo*** **assemblies using uncorrected and corrected reads**

	**Uncorrected**				**Corrected**		
**Assembly**	**Scaffolds**	**N50 (kb)**	**NGA50 (kb)**		**Scaffolds**	**N50 (kb)**	**NGA50 (kb)**
*E. coli*	2,120	31	84		1,840	37	85
*S. aureus*	737	192	145		603	1,771	202

Assemblies of uncorrected and corrected reads using Velvet. *E. coli* reads are paired and assembled using default parameters. *S. aureus* reads are comprised of paired fragment reads with average inserts of length 180 and short jump reads with average inserts of length 3500. These reads are assembled using parameterization as described in GAGE.

### Performance

Pollux requires 3, 5, and 24 minutes when correcting MiSeq, GS Junior (1), and PGM (2) *E. coli* data sets, respectively, when executed on a 8 core Linux machine with an Intel Core i7-3820 (3.60 gigahertz) processor and 64 GB of memory (maximum memory requirements were 9.7 GB). The Ion Torrent PGM *E. coli* data sets had significantly more errors reported than the similar MiSeq and GS Junior data sets. Pollux uses a maximum of 1 GB of memory when counting *k*-mers and correcting the Illumina MiSeq data set [3] containing 19M distinct *k*-mers and 250M bases. We conduct a further test of memory and execution time requirements by running Pollux on the GAGE [2] human chromosome 14 data set (http://gage.cbcb.umd.edu/data/) consisting of 61.5 million paired-end reads total. This correction requires 6.5 hours and uses a peak of 30 GB of memory during *k*-mer counting and 23 GB of memory during correction.

### Filtering

We note that our approach to filtering reads based on unique *k*-mers is an improvement over a naive quality score approach. To verify this we compared the effect of removing reads using both our software’s unique *k*-mer approach with a simple quality score approach. The unique *k*-mer approach is accomplished using our error correction software. Reads that contain more than 50% unique *k*-mers after attempting correction are removed. The quality score approach is accomplished using a custom Python script. The script removes reads which contain more than 10% low quality bases, as reported by the sequencer. We define low quality bases to be a Phred [20] quality score of Q10 or less. We then consider the reads which are removed by the quality score approach but not by the *k*-mer approach. These are reads that are designated as having poor quality scores, but which our error correction software considers valuable. We find that our error correction software is capable of correcting many of the errors in these reads despite having an abundance of low quality scores. The most notable difference is with respect to the Ion Torrent PGM (1) *E. coli* data set. We find that our software is able to correct 87% of the 3.3M errors found in reads that would be discarded exclusively through a simple quality score approach. Our software’s *k*-mer based removal approach can evaluate the usefulness of a read after attempting corrections, retaining more information than filtering using a simple quality based approach before correction.

## Discussion

The number and kinds of reported corrections, both by our error correction software and by our alignment evaluation procedure, corresponds with what we would expect to correct for the respective sequencing technologies and with the results reported by Loman *et al.*. With the *E. coli* data set, Pollux reports 98% of attempted MiSeq corrections as substitution, 90% of PGM(1) as indel, and 92% of GS Junior (2) as indel. Similarly, our alignment evaluation procedure reports 99% of successful MiSeq corrections as substitution, 90% of PGM (1) as indel, and 93% of GS Junior (2) as indel. With respect to GS Junior (2) corrections, insertion corrections appear to be more frequent at 70% than deletions at 23%, as reported by our evaluation procedure. We report per base indel corrections at 1.9% for PGM (1), 0.33% for GS Junior (2), and 0.0021% for MiSeq. This agrees with Loman *et al.* who report per base indels at 1.5% for PGM (1), 0.38% for GS Junior (2), and 0.001% for MiSeq. Similarly, we report per base substitution corrections at 0.1% for MiSeq which agrees with Loman *et al.* at 0.1%.

**Figure 3:**
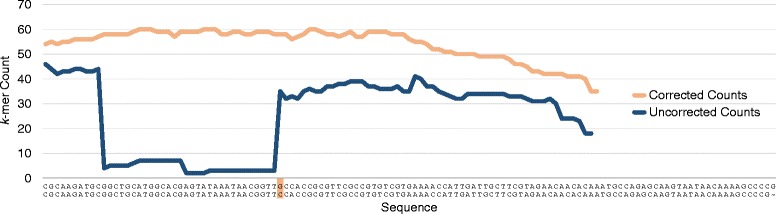
**The** ***k*** **-mer counts associated with the uncorrected and corrected version of an Ion Torrent PGM read containing a highlighted deletion error.** As is common with PGM data, the counts associated with the deletion error are not all reduced to one. This is a result of multiple similar deletion errors coinciding. The overall depth of the read increases after correction, suggesting a large number of errors are removed in other reads.

An example of the changes in *k*-mer counts before and after correction is provided in Figure 3. The effect is most noticeable when correcting Ion Torrent PGM reads. The *k*-mers associated with the uncorrected read reveal one of the difficulties with the data: errors are often not unique and instead frequently coincide, resulting in erroneous regions with *k*-mer counts that do not drop to one. This is problematic because a read containing an accurate length homopolymer will contain a *k*-mer count drop relative to the number of errors in other reads containing the same homopolymer region. We are therefore required to have a more forgiving correction procedure for homopolymers which allows for accurate length homopolymers that otherwise appear erroneous. When the *k*-mers are re-evaluated after all corrections, we discover the overall coverage of the read has increased significantly and *k*-mer counts across reads become more reflective of a random sampling of the genome, as many errors have been corrected. The average *k*-mer counts also increase in MiSeq and GS Junior corrected reads. However, the effect is much less extreme.

While the current version of the software is effective, there are some areas that may be improved by future refinements. The number of corrected errors could be improved by targeting adjacent errors and other coincident errors that are not homopolymers. Within the homopolymer correction algorithm, we do not correct errors other than homopolymer repeats and thereby ignore all other multinucleotide errors. Correcting these errors would require exploring a larger space of correction possibilities. There is also room for improvement when correcting the rarer error types in sequencing technologies. For example, we appear to be slightly overcorrecting substitution errors in GS Junior reads and potentially under-correcting indel errors in MiSeq reads. Additionally, we have a somewhat lower success rate correcting deletion errors than insertion errors across all technologies. This may be a consequence of deletions resulting in fewer *k*-mer count evaluations and therefore simpler to correct. The running time and memory requirements may be improved by incorporating dedicated *k*-mer counting software, such as BFCounter [21], Turtle [22], or KMC [23].

We believe that our success correcting a mixed data set lends evidence to our correction software’s ability to correct more complicated mixed data sets such as metagenomic data. This is supported by our successful correction of *E. coli* MiSeq data which contains 25-fold chromosome coverage and 625-fold phage coverage, suggesting our software is able to correct the majority of errors in the presence of highly variable coverage.

## Conclusion

The *k*-mer count approach used by our software is highly effective at correcting errors across different sequencing platforms, including Illumina MiSeq, Roche 454 GS Junior, and Ion Torrent PGM data sets. These corrections are sequencing technology appropriate and agree with published findings. Our software is sensitive to low-depth sequencing regions and can correct errors in the presence of highly variable coverage while introducing few new errors. Additionally, we find our software corrects the majority of errors in a mixed genome environment. We believe our software is a versatile tool that may be used in a variety of applications.

## Availability

The source code for Pollux is distributed freely.**Project name:** Pollux**Project home page:**http://github.com/emarinier/pollux**Operating system(s):** Unix-based 64-bit OS**Programming language:** C**Other requirements:** None**License:** GNU GPL**Any restrictions to use by non-academics:**Non-academics may freely use this software.
